# Functional characterization of DYRK1A missense variants associated with a syndromic form of intellectual deficiency and autism

**DOI:** 10.1242/bio.032862

**Published:** 2018-04-15

**Authors:** Esti Wahyu Widowati, Sabrina Ernst, Ralf Hausmann, Gerhard Müller-Newen, Walter Becker

**Affiliations:** 1 Institute of Pharmacology and Toxicology, Medical Faculty, RWTH Aachen University, 52074 Aachen, Germany; 2 Chemistry Study Program, Faculty of Science and Technology, State Islamic University (UIN) Sunan Kalijaga, Yogyakarta 55281, Indonesia; 3 Institute of Biochemistry and Molecular Biology, Medical Faculty, RWTH Aachen University, 52074 Aachen, Germany

**Keywords:** DYRK1A, Haploinsuffiency, Intellectual disability, Missense mutation, MRD7

## Abstract

Haploinsufficiency of *DYRK1A* is a cause of a neurodevelopmental syndrome termed mental retardation autosomal dominant 7 (MRD7). Several truncation mutations, microdeletions and missense variants have been identified and result in a recognizable phenotypic profile, including microcephaly, intellectual disability, epileptic seizures, autism spectrum disorder and language delay. DYRK1A is an evolutionary conserved protein kinase which achieves full catalytic activity through tyrosine autophosphorylation. We used a heterologous mammalian expression system to explore the functional characteristics of pathogenic missense variants that affect the catalytic domain of DYRK1A. Four of the substitutions eliminated tyrosine autophosphorylation (L245R, F308V, S311F, S346P), indicating that these variants lacked kinase activity. Tyrosine phosphorylation of DYRK1A-L295F in mammalian cells was comparable to wild type, although the mutant showed lower catalytic activity and reduced thermodynamic stability in cellular thermal shift assays. In addition, we observed that one variant (DYRK1A-T588N) with a mutation outside the catalytic domain did not differ from wild-type DYRK1A in tyrosine autophosphorylation, catalytic activity or subcellular localization. These results suggest that the pathogenic missense variants in the catalytic domain of DYRK1A impair enzymatic function by affecting catalytic residues or by compromising the structural integrity of the kinase domain.

This article has an associated First Person interview with the first author of the paper.

## Abstract (precis)

**Summary:** We have analyzed the functional consequences of amino acid substitutions in the protein kinase DYRK1A that have been identified as pathogenic in patients with microcephaly, intellectual disability and autism.

## INTRODUCTION

DYRK1A is a member of the DYRK [dual-specificity tyrosine (Y) phosphorylation-regulated kinase] family of protein kinases which play important roles in the regulation of cell differentiation, proliferation, and survival (Becker and Joost, 1999; Aranda et al., 2011). DYRKs autophosphorylate a critical tyrosine residue in the activation loop (Y321 in DYRK1A) (Kentrup et al., 1996). Tyrosine autophosphorylation is a one-time event coupled to protein folding and maturation and is necessary for achieving full catalytic activity (Lochhead et al., 2005). The active mature DYRKs then phosphorylate serine and threonine residue in a large variety of nuclear and cytoplasmic protein substrates (Aranda et al., 2011; Becker and Sippl, 2011; Tejedor and Hämmerle, 2011). DYRK1A harbors a functional nuclear localization signal and a polyhistidine sequence that targets the protein to the splicing factor compartment (Alvarez et al., 2003).

The function of *DYRK1A* is highly dependent on its gene dosage (Duchon and Herault, 2016). Human *DYRK1A* was identified as a Down syndrome candidate gene because it is located in the Down syndrome critical region on chromosome 21 (Guimerá et al., 1996). Overexpression of DYRK1A is considered a major cause of the neurodevelopmental alterations that underlie altered brain function in Down syndrome (Tejedor and Hämmerle, 2011; Duchon and Herault, 2016). Decreased expression due to haploinsufficiency of *DYRK1A* is also pathogenic and causes a developmental syndrome termed mental retardation autosomal dominant 7 (MRD7) (OMIM 614104). Patients with heterozygous truncation mutations or deletion of *DYRK1A* share a phenotype with microcephaly, intellectual disability, language delay, epileptic seizures, neonatal feeding problems, and facial dysmorphism (Møller et al., 2008; Courcet et al., 2012; Ji et al., 2015; van Bon et al., 2011; Redin et al., 2014; Ruaud et al., 2015; Luco et al., 2016). Pathogenic *de novo* mutations in the *DYRK1A* gene were identified in 19 of 4293 patients in the Deciphering Developmental Disorder (DDD) Study and account for around 0.5% of syndromic intellectual disability in this cohort (Evers et al., 2017). *DYRK1A* mutations were also observed in exome sequencing studies of patients with autism spectrum disorder (ASD) (O'Roak et al., 2012a,b; Samocha et al., 2014). Autistic behavior is now recognized as a component of the core phenotype of the *DYRK1A* haploinsufficiency syndrome (van Bon et al., 2016; Bronicki et al., 2015; Ji et al., 2015; Earl et al., 2017; Dang et al., 2018).

Substantial experimental evidence supports a key role of DYRK1A as a regulator of brain growth and function, including neurogenesis, neuronal proliferation and differentiation, synaptic transmission as well as neurodegeneration (Murakami et al., 2006, 2009; Hämmerle et al., 2011; Tejedor and Hämmerle, 2011; Wegiel et al., 2011; Guedj et al., 2012; Park and Chung, 2013; Bellmaine et al., 2017). Mutations in the orthologous *Drosophila* gene (*mnb*) result in a ‘minibrain phenotype’ of adult flies with reduced optic lobes and central brain hemispheres (Tejedor et al., 1995). Similarly, mice with only one functional copy of the *Dyrk1a* gene show a reduction of brain volume in a region-specific manner and display impaired cognitive flexibility and social contacts as well as susceptibility to hyperthermia-induced seizures (Fotaki et al., 2002; Raveau et al., 2018).

Most cases of *DYRK1A* haploinsufficiency are caused by protein disrupting mutations such as microdeletions and truncating single nucleotide variants. Several missense variants have also been identified, most of which map close to the catalytic loop or the substrate binding site. Structural considerations suggest that these variants interfere with the protein kinase activity of DYRK1A (Ji et al., 2015; Evers et al., 2017). Nevertheless, the pathogenic DYRK1A missense variants have not yet been functionally analyzed.

In this study, we analyzed the functional consequences of the disease-causing missense variants of DYRK1A. Most mutations abolished kinase activity, while one variant displayed only partial decrease in enzymatic activity and a reduction of thermal stability.

## RESULTS

### Most DYRK1A missense mutations in MRD patients affect the catalytic domain

**Fig. 1.**
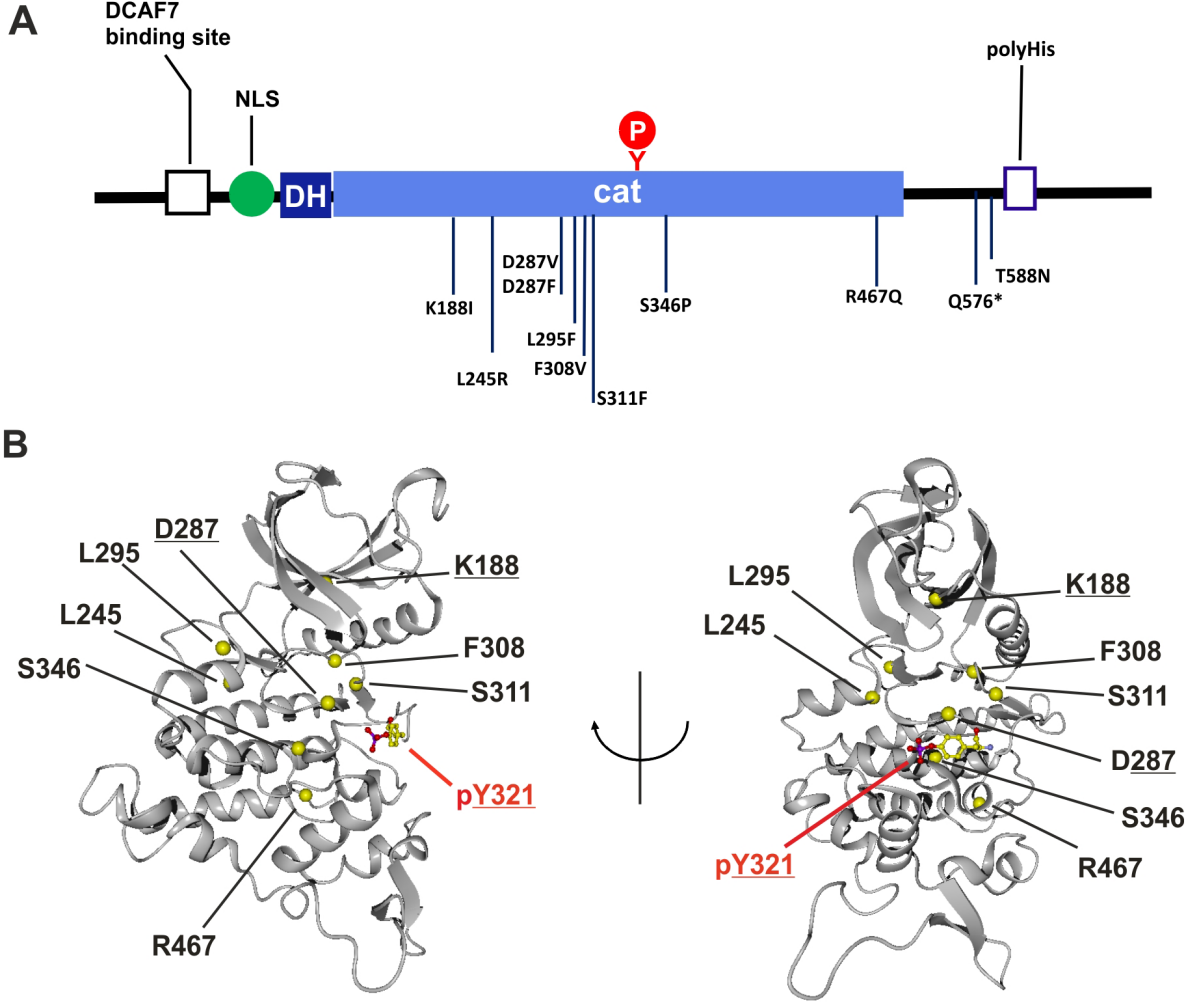
**Structure of DYRK1A and location of pathogenic missense mutations.** (A) Domain organization of DYRK1A. Sequence characteristic in the N-terminal domain include a binding site for the adaptor protein DCAF7, a nuclear localization sequence (NLS) and the DYRK homology box (DH). Autophosphorylation of Y321 in the catalytic domain (cat) is critical for full catalytic activity. The carboxyl terminal region harbors a track of 13 histidines that targets the protein to the nuclear speckle compartment. (B) Ribbon representation of the kinase domain, with one orientation rotated 90° around the depicted axis relative to the other (PDB entry 2VX3). The Cα positions of the amino acids that are affected in the pathogenic variants are highlighted by yellow balls. The autophosphorylated pY321 is shown as a ball-and-stick model. Underlined amino acids (K188, D287) have previously been functionally studied by analysis of point mutants.

To understand how MRD7-linked mutations affect the function of DYRK1A, we compiled a list of pathogenic *DYRK1A* missense mutations from published reports and the ClinVar and Decipher genome resources (Table 1). Most substitutions are located in the kinase domain (Fig. 1A), clustering in the proximity of the ATP binding pocket and the catalytic center (Fig. 1B).

**Table 1.**
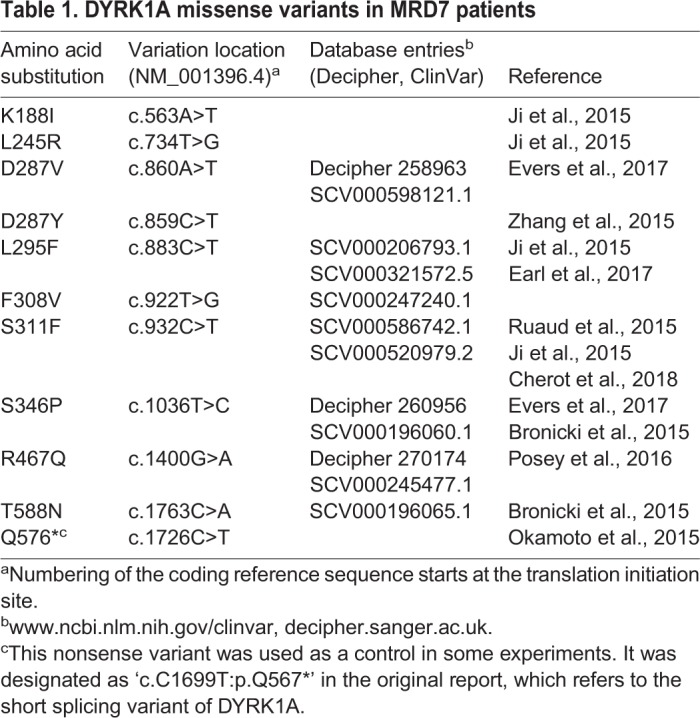
**DYRK1A missense variants in MRD7 patients**

Three variants affect amino acids that are essential for the catalytic function of the kinase. K188 is involved in the binding of the co-substrate ATP, and D287 is the catalytic residue required for phosphate transfer. Substitution of either K188 or D287 is already known to render DYRK1A catalytically inactive (Kentrup et al., 1996; Walte et al., 2013). In the present study, we focused on the other missense mutations (L245R, L295F, F308V, S311F, S346P and R467Q) to explore the structure-function relationship of the DYRK1A catalytic domain.

L245 is located in the hinge region that connects the N- and the C-terminal lobe of the catalytic domain. Based on molecular modelling analysis, Ji et al. (2015) have proposed that the substitution of leucine by the larger side chain of arginine generates steric clashes that may disrupt the integrity of the active site and prevent ATP binding.

L295 is a component of the hydrophobic ‘C-spine’ (catalytic) in the core of the kinase domain (Kornev et al., 2008; Taylor and Kornev, 2011). This series of hydrophobic residues organizes the elements of the catalytic domain to ensure their precise positioning in the active conformation. The replacement of L295 by phenylalanine may destabilize the domain structure by altering the geometry of the C-spine.

F308 is a key component of the hydrophobic ‘R-spine’ (regulatory) that is a hallmark signature of every active kinase conformation (Meharena et al., 2013). This structural motif consists of a non-contiguous ensemble of hydrophobic residues that traverses both lobes of the kinase core. Moreover, F308 belongs to the highly conserved DFG motif and forms the link between one hydrophobic residue from the N-lobe and one residue from the C-lobe.

In contrast to the DFG motif, the neighboring amino acids (S310-S311-C312) at the beginning of the activation loop are distinctive for the DYRK family of protein kinases. C312 can form an intramolecular disulfide bridge with a cysteine in the catalytic loop (C286) that is conserved in all members of the DYRK family (Becker and Sippl, 2011; Alexeeva et al., 2015). The replacement of S311, which forms a hydrogen bond with the carbonyl of H285, by the spacious and hydrophobic phenylalanine residue is likely to disrupt the relative positioning of the activation loop and the catalytic loop if not the entire domain fold.

S346 is located in the middle of the F-helix, which acts as a central scaffold of the C-lobe (Taylor and Kornev, 2011). As already noted by Evers et al. (2017), the presence of proline distorts the F-helix and thereby affects the positioning of the other structural elements in the C-lobe.

The C-terminal part of the kinase domain (helices G, H and I comprising amino acids 360-479 in DYRK1A) is rather far from the substrate and ATP-binding sites and is rarely affected by disease-causing SNPs (Torkamani et al., 2008). However, R467 is a conserved residue in the eukaryotic protein kinase family that is engaged in a salt bridge interaction with E331 at the end of the activation loop (Soundararajan et al., 2013). This interaction is an invariant feature of the conserved protein kinase fold (Yang et al., 2012).

For comparison, we included one variant that results in the substitution of a residue in the non-catalytic C-terminal domain and was also classified as likely pathogenic (T588N, Bronicki et al., 2015). T588 is located in close proximity to the polyhistidine repeat in the C-terminal part of DYRK1A. It is not known whether this region folds into a defined three-dimensional structure.

### Tyrosine autophosphorylation of DYRK1A missense mutants

To examine the consequences of the pathogenic missense mutations on the function of DYRK1A, we introduced the respective amino acid substitutions into a GFP-DYRK1A fusion protein. Wild-type and mutant constructs were immunoprecipitated from transiently transfected HEK293 cells and subjected to immunoblot analysis to determine the phosphorylation of Y321. Autophosphorylation of the tyrosine in the activation loop is a key step in the maturation of DYRKs, and we used the phosphorylation status of Y321 as an indicator of correct folding and conformational integrity of DYRK1A. For comparison, we included two variants that do not affect the catalytic domain, the T588N missense and the Q576* nonsense variant. In addition, GFP-DYRK1A-K188R and D287N served as catalytically inactive controls, and DYRK1A-Y321F was used as a control for antibody specificity.

**Fig. 2.**
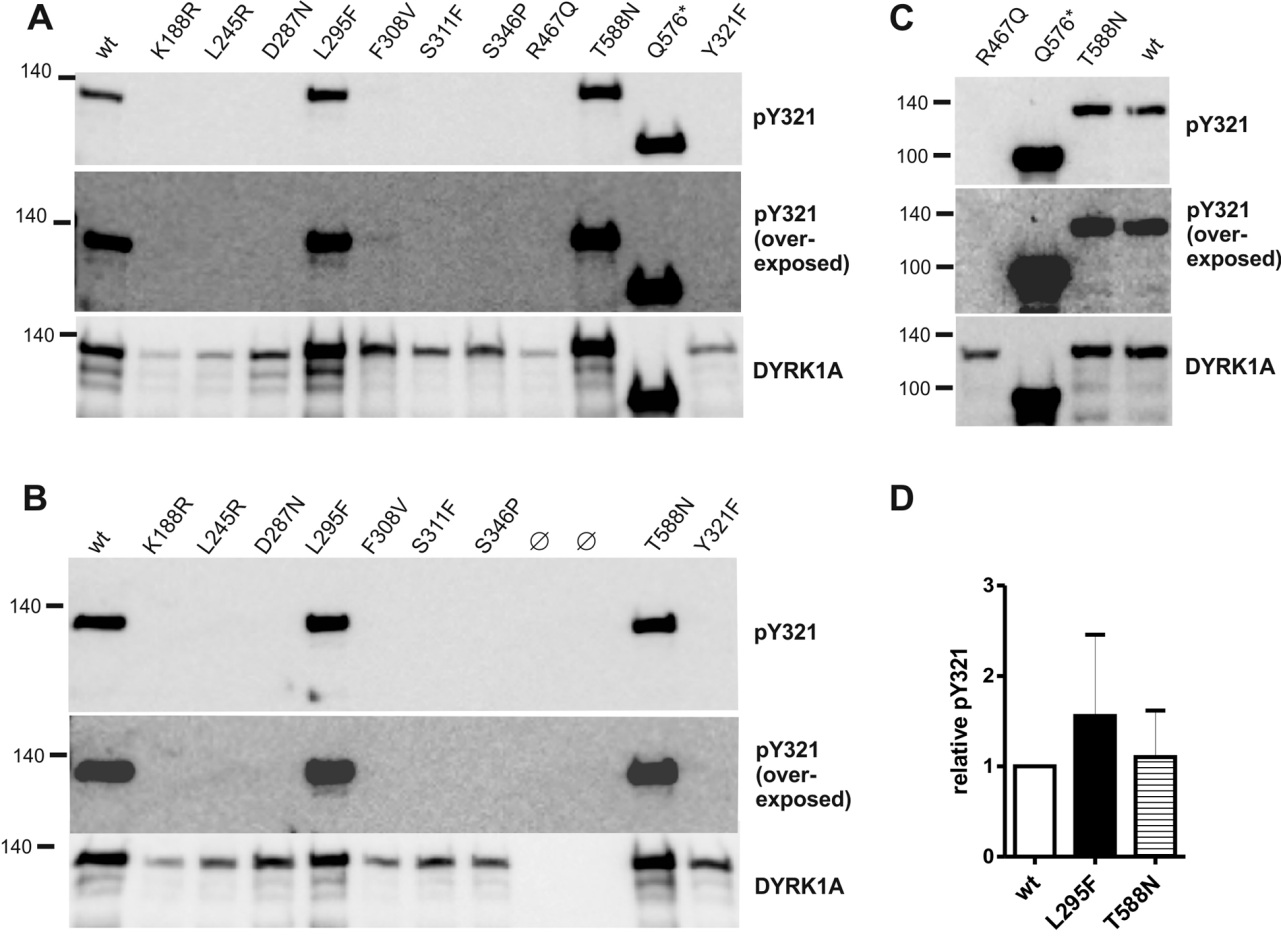
**Tyrosine autophosphorylation of wild-type and variant DYRK1A constructs.** Wild-type (wt) and mutant GFP-DYRK1A fusion proteins were immunoprecipitated with a GFP antibody from cell extracts of transiently transfected HEK293 cells and subjected to immunoblot analysis. Tyrosine phosphorylation of the activation loop (Y321) was analyzed using an antibody against the activation loop tyrosine in HIPK2 that cross-reacts with the corresponding residue in DYRK1A (Abu Jhaisha et al., 2017). Relative tyrosine phosphorylation was calculated ratio of the pY321 signal relative to total amounts DYRK1A constructs as detected with a DYRK1A specific antibody. (A-C) Representative western blots from selected experiments, including overexposed images to demonstrate the absence of pY321 in the weakly expressed variants. (D) Quantitative evaluation of the catalytically active missense variants (means and s.d.; *n*=5 for wt versus L295F and *n*=6 for wt versus T588N)**.** Differences were not statistically significant by one-sample *t*-test (*P*>0.05).

Fig. 2 shows that all amino acid exchanges in the kinase domain of DYRK1A resulted in a complete loss of tyrosine autophosphorylation, except for L295F. Furthermore, several of the mutant proteins were expressed at reduced levels as compared with wild-type DYRK1A, suggesting that these substitutions affect protein stability. In particular, R467Q could be adequately evaluated in only one experiment (Fig. 2C), so that our observation of absent tyrosine phosphorylation must be considered preliminary. Given that the phosphorylation of Y321 is an essential step in the maturation of DYRK1A, we classified L245R, F308V, S311F, S346P as loss-of-function mutants and did not further investigate the properties of these proteins. In contrast, quantitative evaluation confirmed that the phosphotyrosine content of L295F was not significantly different from wild-type DYRK1A or T588N (Fig. 2D), indicating that this variant is an active protein kinase.

### Kinase activity

**Fig. 3.**
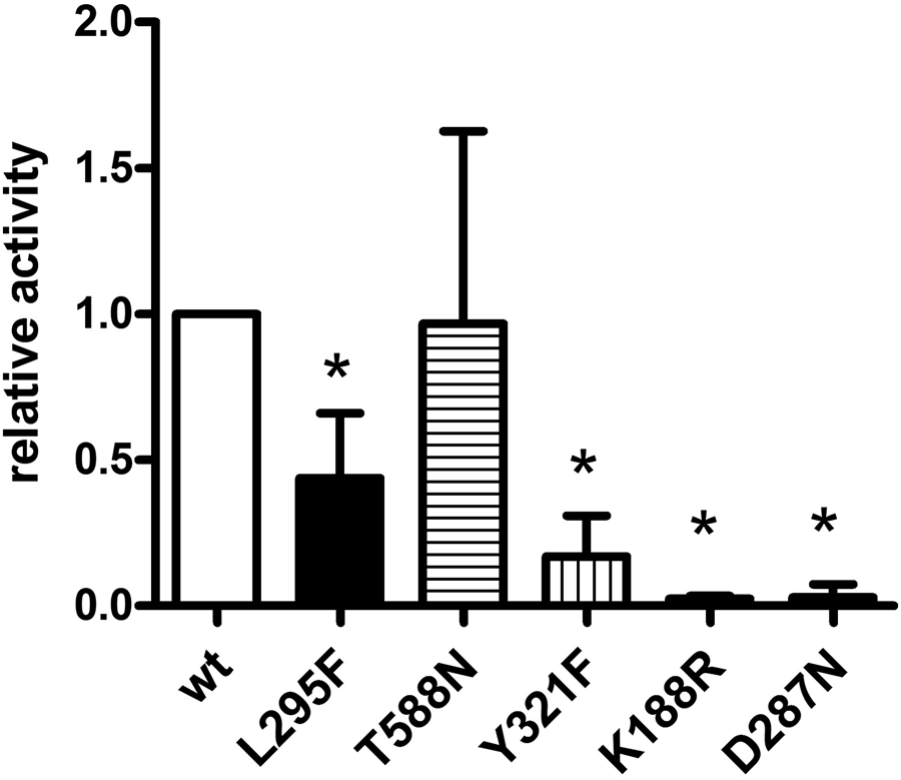
**Effects of mutations on DYRK1A kinase activity.** Wild-type (wt) and mutant GFP-DYRK1A fusion proteins were immunoprecipitated with a GFP antibody from cell extracts of transiently transfected HEK293 cells and subjected to radiometric immunocomplex kinase assays with the peptide substrate, DYRKtide. *In vitro* kinase activities were normalized to the amount of the respective GFP-DYRK1A protein as quantified by immunoblot analysis. Results are shown relative to wt DYRK1A (means and s.d.; *n*=5 for T588N, *n*=4 for L295F and D287N and *n*=3 for Y321F and K188R). Pairwise differences between wt and variants were tested for statistical significance by one-sample *t*-test (*P<0.05).

Next we determined the enzymatic activity of DYRK1A-L295F and T588N towards an exogenous substrate. GFP-DYRK1A constructs were immunoprecipitated from HEK293 cells and subjected to kinase assays using the peptide substrate, DYRKtide. DYRK1A-Y321F was included as a control with strongly reduced but detectable catalytic activity (Himpel et al., 2001). Consistent with their capacity of tyrosine autophosphorylation, DYRK1A-L295F and T588N were found to be active kinases (Fig. 3). Notably, DYRK1A-L295F showed significantly lower catalytic activity than wild-type DYRK1A.

### Reduced conformational stability of DYRK1A-L295

**Fig. 4.**
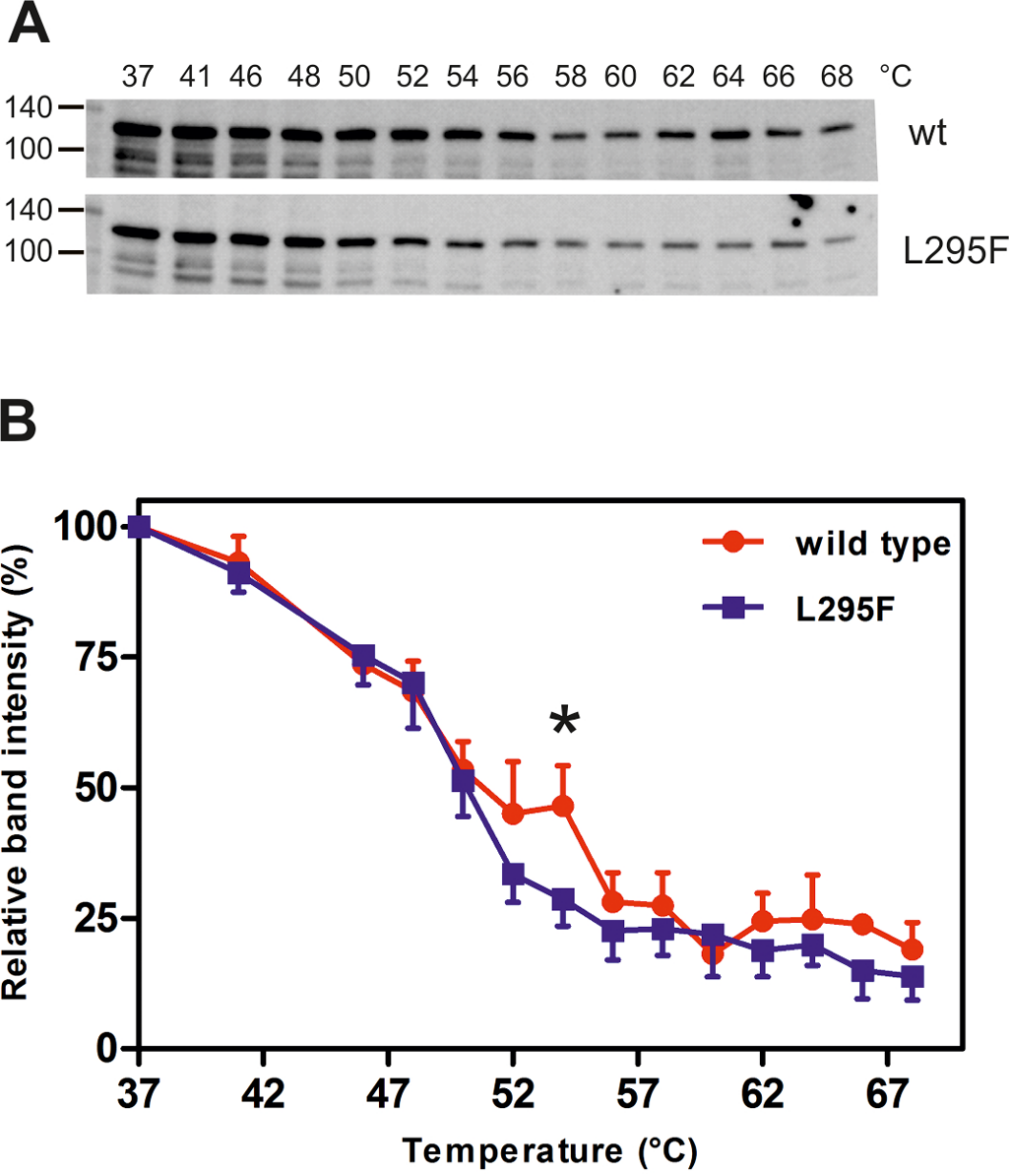
**Thermal stability assay.** HEK293 cells stably expressing wild-type (wt) DYRK1A or L295F mutant were harvested and resuspended with PBS. Multiple aliquots of the cell suspensions were subjected to heat treatment for 3 min as indicated. Cells were lysed using Triton X-100 and cleared by centrifugation. The presence of the target protein in the soluble fraction was detected using western blot analysis and quantified. (A) Blots from a representative experiment. (B) Quantitative evaluation of four experiments (means and s.d.). For each pair of data points, differences in the amount of soluble protein were tested for statistical significance by one-sample *t*-test (*P<0.05).

Our results show that the substitution of L295 by phenylalanine still allows the kinase domain to obtain an active conformation. To assess whether the amino acid exchange compromised the conformational stability of the kinase domain, we used a modified version of the cellular thermal shift assay (CETSA) (Jafari et al., 2014). To establish melting curves, suspensions of HEK293 cells expressing wild-type or mutant GFP-DYRK1A were aliquoted and heated to different temperatures. After cooling, cells were lysed with the help of non-ionic detergent and soluble fractions were separated from denatured, precipitated proteins by centrifugation. As shown in Fig. 4, denaturation of DYRK1A occurred gradually over a wide range of temperatures. The melting point of about 50°C is close to the value of 52°C that was previously reported for endogenous DYRK1A (Zhou et al., 2017). The L295F substitution favored protein denaturation at temperatures around the melting point, suggesting that this mutation compromised the conformational stability of the catalytic domain.

### Impaired tyrosine autophosphorylation of DYRK1A-L295F in a cell-free translation system

**Fig. 5.**
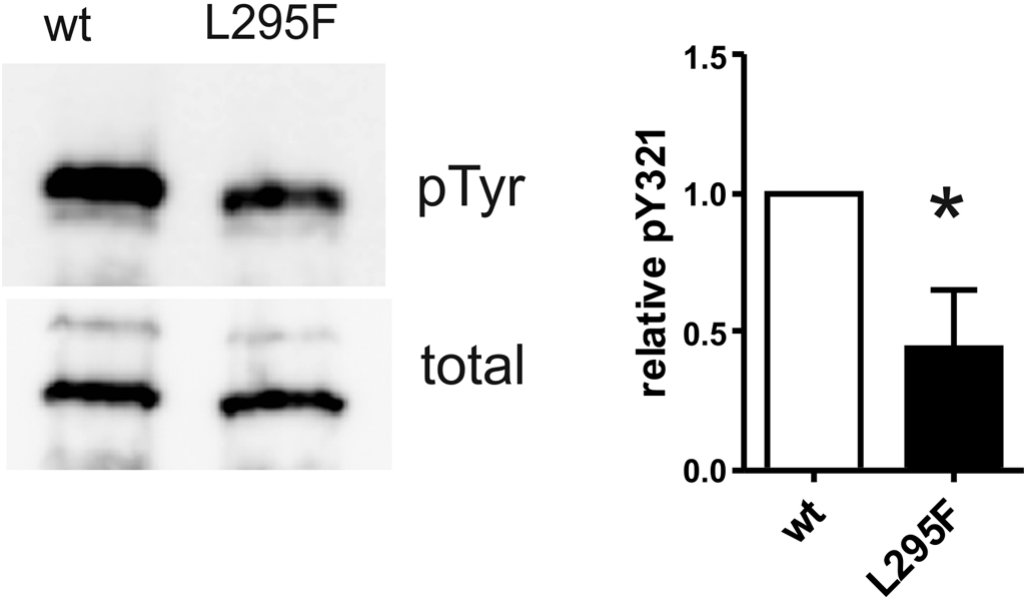
**Reduced tyrosine autophosphorylation of *in vitro*-translated DYRK1A-L295F.** DYRK1A constructs (28-499) were expressed in a cell-free *E. coli-*derived expression system. Coupled *in vitro* transcription-translation reactions were incubated for 90 min at 37°C before reaction products were subjected to immunoblot analysis. For quantification, pTyr signals were normalized to the total amounts of recombinant DYRK1A as determined by detection of the N-terminal StrepTag (means and s.d., *n*=3). The difference in relative tyrosine autophosphorylation was tested for statistical significance by one-sample *t*-test (*P<0.05).

DYRK1A is able to attain its active, autophosphorylated conformation autonomously in the absence of co-factors or chaperones (Walte et al., 2013). We expressed wild-type DYRK1A and DYRK1A-L295F in a bacterial *in vitro* translation system to assess whether the amino acid exchange affected tyrosine autophosphorylation in a cell free system. Under these conditions, tyrosine autophosphorylation of DYRK1A-L295F was significantly reduced as compared to the wild-type enzyme (Fig. 5).

### Subcellular localization of the DYRK1A variants

**Fig. 6.**
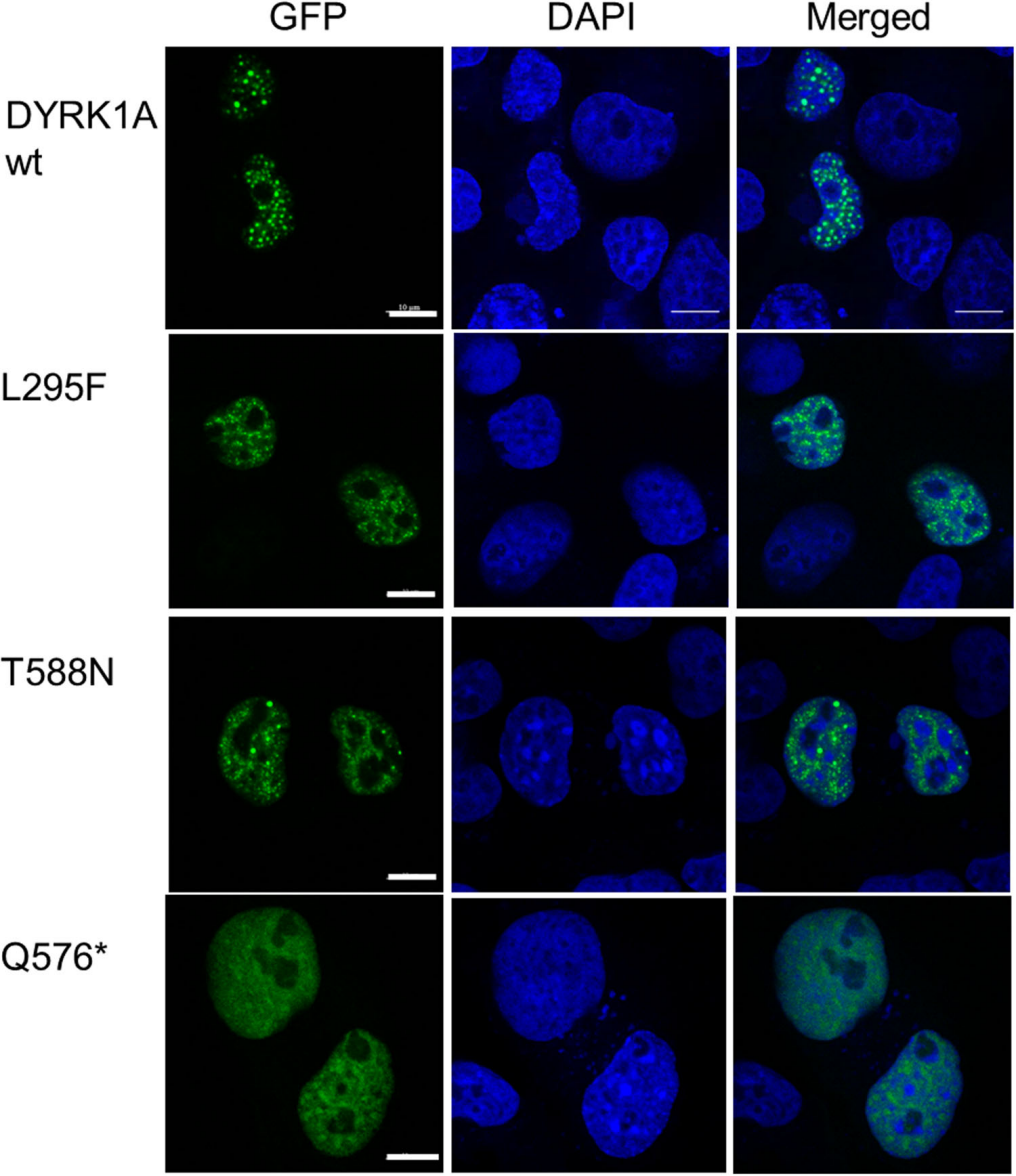
**Subcellular localization of wild-type and mutant GFP-DYRK1A.** COS7 cells were transiently transfected with expression vector for DYRK1A wild-type, L295F, T588N, or Q576*. The cellular localization of GFP-DYRK1A constructs was evaluated by GFP autofluorescence in relation to DAPI for nuclear staining. Representative images from four experiments are shown. At least 100 transfected cells were evaluated for each construct. Scale bars: 10 µM.

The cellular function of DYRK1A depends on its localization in the cell. To reveal whether the pathogenic variants affected the subcellular distribution of DYRK1A, we expressed GFP-DYRK1A constructs in COS-7 cells. This cell line was chosen because the large, flat cellular shape of these cells facilitates microscopic analysis (Alvarez et al., 2003). Most cells expressing wild-type GFP-DYRK1A showed a punctate pattern in the nucleus (Fig. 6), consistent with previous observations that DYRK1A is localized to nuclear speckles that constitute the splicing factor compartment (Becker et al., 1998; Alvarez et al., 2003). There was no detectable difference in the pattern of distribution between wild-type DYRK1A and the L295F and T588N point mutants. In contrast, DYRK1A-Q567* did not accumulate in speckles, although nuclear localization was maintained, because the polyhistidine-containing region between amino acids 590 and 616 in DYRK1A has been identified as the speckle-targeting sequence (Alvarez et al., 2003).

## DISCUSSION

The present study describes the functional analysis of DYRK1A missense variants that were reported as pathogenic in patients with a syndromic form of autism and intellectual disability (MRD7). Most cases of MRD7 are caused by deletion of the *DYRK1A* gene or disruptive mutations that cause a loss of function (Møller et al., 2008; van Bon et al., 2016; Ji et al., 2015; Luco et al., 2016; Earl et al., 2017). Therefore, the missense mutations were expected to interfere with important functions of DYRK1A. Indeed, two of the affected amino acids (K188, D287) had already been shown to be essential for the enzymatic activity of DYRK1A. Here we found that another 5 substitutions (L245R, F308V, S311F, S346P and R467Q) also eliminate tyrosine autophosphorylation of DYRK1A, indicating that these variants lack catalytic activity. Interestingly, one missense variant in the catalytic domain (L295) had a more subtle effect on protein function, while we observed no functional effect of the T588N mutation in the C-terminal region.

The complete lack of phosphotyrosine in most of the DYRK1A mutants shows that the catalytic domain cannot attain a conformation capable of ATP binding and/or phosphoryl transfer. F308, S346 and R467 have conserved structural functions in the kinase domain fold (see above) and are hotspots of disease-causing mutations in the protein kinase superfamily (Torkamani et al., 2008). Intriguingly, L245 and S311 are more variable in the kinase superfamily but are well conserved in the DYRK family (Becker and Joost, 1999).

In contrast to the other amino acid exchanges in the catalytic domain, L295F did not impair the autophosphorylation of Y321 in mammalian cells. Interestingly, a patient carrying this allele seemed to display a relatively mild phenotype, and was not affected by feeding difficulties, seizures or intellectual disability at the age of 21 months (Ji et al., 2015). Nevertheless, marked microcephaly, dysmorphic features, abnormal gait and stereotypies were observed, and the pathogenicity of the variant was supported by the identification of a second patient (Earl et al., 2017). This mutation destabilized the domain fold in thermal stability assays and reduced catalytic activity *in vitro*. Furthermore, DYRK1A-L295F showed decreased tyrosine autophosphorylation in a bacterial *in vitro* translation system. It seems plausible that chaperones can partially compensate for the thermodynamic instability of the kinase domain when DYRK1A-L295F is expressed in mammalian cells. It is also possible that a fraction of DYRK1A-L295F molecules fails to autophosphorylate in HEK293 cells but escapes detection because it is eliminated by the ubiquitin proteasome system.

T588N is the only missense mutation outside the catalytic domain that has been reported to result in a clinical phenotype (Bronicki et al., 2015). This variant did not differ from wild-type DYRK1A in its catalytic activity or subcellular localization. A patient carrying this variant exhibited the cardinal features of MRD7 (intellectual disability, absent or delayed speech, microcephaly and seizures), except that feeding difficulties were not reported (Bronicki et al., 2015). However, it must be noted that another missense variant of this codon (T588P, dbSNP:rs149948846) is present in 21 of the >60,000 reference genomes in the Exome Aggregation Consortium (ExAC) database (http://exac.broadinstitute.org), which excludes individuals affected by severe pediatric disease (Lek et al., 2016). In contrast, no missense alleles corresponding to any of the other pathogenic variants were found in this database. We think that the pathogenicity of the T588N variant requires further confirmation.

Neither of the catalytically active missense variants (L295F and T588N) was detectably mislocalized when the GFP-DYRK1A fusion proteins were ectopically expressed in COS-7 cells. This experimental model system was previously used to characterize the nuclear localization signal in the N-terminal domain and the speckle-targeting function of the polyhistidine track in the C-terminus of DYRK1A (Becker et al., 1998; Alvarez et al., 2003). Nevertheless, it must be noted that the subcellular distribution of endogenous DYRK1A is rather complex and DYRK1A has many extra-nuclear functions. For instance, the majority of endogenous DYRK1A in HeLa cells was found in the cytosolic fraction, while in both human and mouse brain almost 80% of DYRK1A was bound to the cytoskeleton (Kaczmarski et al., 2014; Di Vona et al., 2015). Furthermore, the localization of DYRK1A can be regulated depending on the cell type or developmental state (Hämmerle et al., 2008). The present observation that L295F and T588N do not interfere with the localization of overexpressed DYRK1A to nuclear speckles does not exclude that these substitutions interfere with the participation of DYRK1A in other protein complexes or its association with other subcellular structures.

**Fig. 7.**
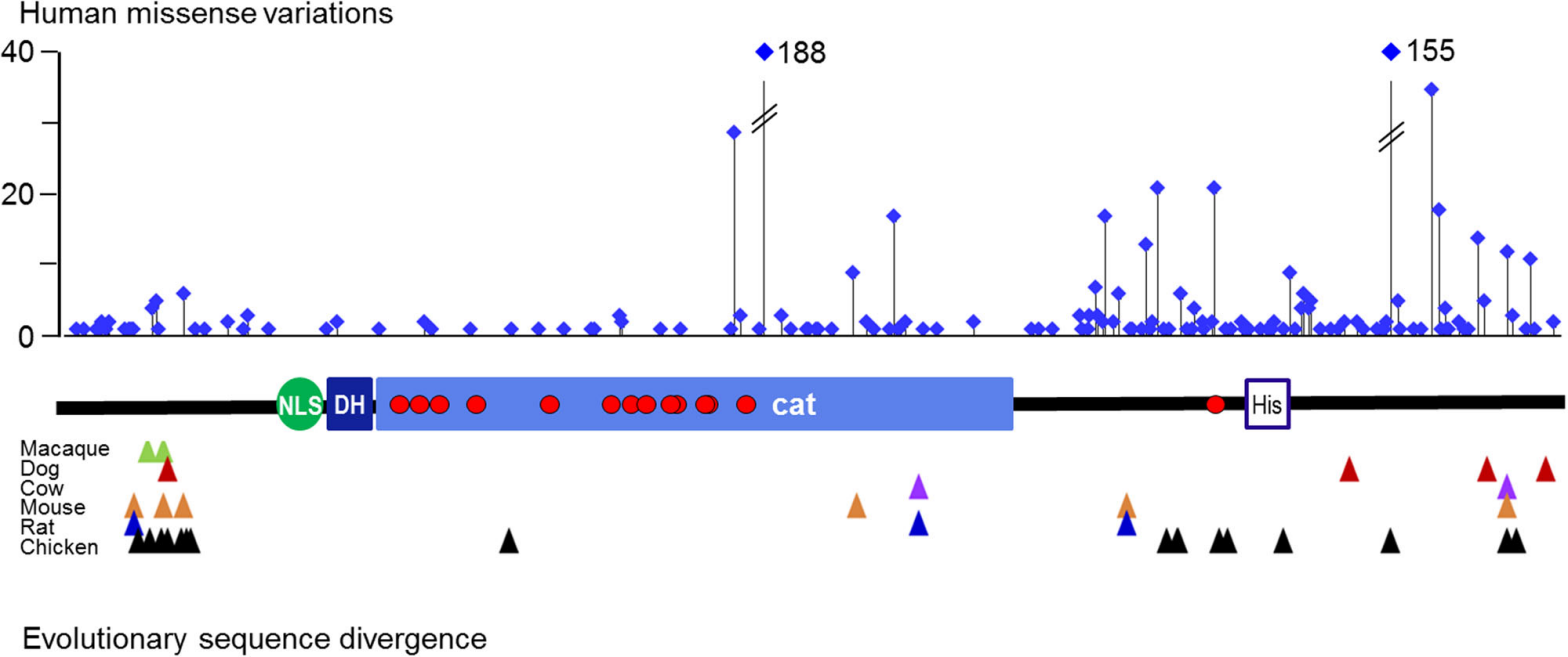
**Sequence variation in DYRK1A.** The graph illustrates the absolute numbers and locations of natural DYRK1A missense alleles in the ExAC database (*n*=60,706 genomes). Numbers of alleles exceeding the limit of the y-axis are written out. *De novo* missense variants classified as ‘pathogenic’ or ‘likely pathogenic’ in the ClinVar database are signified as red circles in the domain diagram of DYRK1A. Differences between the human DYRK1A sequence and orthologs from mammals or chicken are indicated as triangles. This information was deduced from an interspecies multiple sequence alignment in the NCBI Homologene resource (https://www.ncbi.nlm.nih.gov/homologene/55576).

Aside from T588N, all pathogenic missense variants are located in the catalytic domain of DYRK1A and substantially impair enzymatic function either directly by affecting catalytic residues (K188I, D287V) or by compromising the structural integrity of the kinase domain. As previously noted by Evers et al. (2017), non-disease-associated variants show a very different distribution and are depleted in the catalytic domain and the neighboring regions that are also conserved in DYRK1B (amino acids 100-500) (Fig. 7). Similarly, sequence differences between vertebrate DYRK1A orthologues are enriched in the C-terminal domain and a segment in the N-terminal domain. DYRK1A is extremely well conserved in evolution, with only 17 amino acids differing between the chicken and human sequences, which have split with the divergence of reptiles and mammals about 300 million years ago (Kumar and Hedges, 1998). It is interesting that many more missense variants are present in relatively healthy adults than are tolerated between species, because similar evolutionary constraints are expected to act. It has been shown by systematic analysis of 81 proteins domains that genetic variation in the human population generally correlates well with evolutionary conservation (Wiel et al., 2017). We speculate that many of the missense alleles in the human population that do not result in overt developmental disorder may compromise the function of DYRK1A enough to reduce evolutionary fitness and preclude the evolutionary persistence of such variants. Intriguingly, *DYRK1A* is one of the genes that was subject to the strongest selective pressure after introgression of Neanderthal alleles in the gene pools of modern humans (Green et al., 2010; Mozzi et al., 2017).

Several further *DYRK1A* missense variants were described since we initiated our study. Dang et al. (2018) described new DYRK1A variants in patients with autism spectrum disorder and intellectual deficiency, including five missense variants (H119Y, D126Y, R133Q, A195T, L259F). Three of the affected amino acids are located N-terminal of the catalytic domain. Surprisingly, neither of three point mutants that were functionally studied (H119Y, A195T, L259F) differed from wild-type DYRK1A in assays of neurite outgrowth, dendritic spine development and radial migration during mouse cortical development (Dang et al., 2018). This study did not address potential biochemical effects of the substitutions. Unfortunately, the pathogenicity of these variants is not irrevocable, since genetic information about the parents was not available. Evers et al. (2017) report two pathogenic variants (L207P, A277P) that affect residues in proximity of the ATP binding pocket. Guided by structural modelling, the authors hypothesize that these substitution disrupt the overall stability of the kinase domain. It remains to be determined in functional assays whether these variants retain partial activity as we have found for L295F.

## MATERIALS AND METHODS

### Plasmids

Mammalian expression plasmids for wild-type GFP-DYRK1A have been described previously (pEGFP-DYRK1A, Becker et al., 1998; pcDNA5/FRT/TO-GFP-DYRK1A, Sitz et al., 2008). For bacterial *in vitro* translation experiments, partial DYRK1A cDNA encoding for amino acids 28-499 was inserted into the expression plasmid pET-ST2 to create an N-terminal fusion with the Strep-tag 2 sequence (Walte et al., 2013). Point mutants of DYRK1A were generated using the QuikChange method (Stratagene). All mutagenesis plasmids were verified by DNA sequencing. The expression vector for GFP-SF3B1-NT (comprising amino acids 1–492 of human SF3B1) was described before (de Graaf et al., 2006).

### Antibodies

Research Resource Identifiers (RRID) of antibodies are indicated if available (https://scicrunch.org/resources). Rabbit polyclonal antibody against phospho-HIPK2 (pTyr361) was purchased from Thermo Fisher Scientific (#PA5-13045; RRID:AB_10987115) and used at 1:1000 dilution for western blot detection. We have previously shown that this antibody is suitable for detection of pY321 in DYRK1A, due to the sequence similarity of the activation loop in HIPK2 and DYRK1A (Abu Jhaisha et al., 2017). Goat polyclonal antibody against GFP was from Rockland Immunochemicals (Limerick, PA, USA; #600-401-215; RRID:AB_828167) and used at 1:1000. Goat anti-DYRK1A antibody was obtained from Everest Biotech (Upper Heyford, UK; #EB11483) and used at 1:500. Phosphorylation of pT434 in SF3B1 was detected with a custom-made rabbit antibody (de Graaf et al., 2006) using a dilution of 1:200. Samples from untransfected cells were used for antibody validation.

### Cell culture and transfection

HEK293 and COS7 cells were expanded from cryopreserved stocks that were available in the laboratory from previous studies (Becker et al., 1998). Cells were cultured in Dulbecco's modified Eagle medium (DMEM) supplemented with 10% fetal bovine serum (Sigma-Aldrich). Cells were transfected with plasmid DNA using Fugene HD (Promega, Mannheim, Germany). A stable HEK293 Flp-In cell clone with doxycycline-inducible expression of GFP-DYRK1A has been described before (Sitz et al., 2008). An isogenic HEK293 cell line for expression of GFP-DYRK1A-L295F was established by Flp recombinase-mediated integration of the pcDNA5/FRT/TO expression vector to the same locus.

### Immunoprecipitation kinase assay

HEK293 cells were transiently transfected with expression vectors for wild-type or mutant GFP-DYRK1A (pEGFP for WT, D287N, K188R, Y321F and pcDNA5/FRT/TO for L245R, L295F, F308V, S311F, S346P, R467Q, Q576*, T588N). Two days after transfection, cells in a 10-cm dish were washed with cold PBS and lysed using 1 ml non-denaturing buffer (50 mM Tris-Cl pH 7.5, 150 mM NaCl, 2 mM EDTA, 0.5% Igepal CA-630, supplemented with 1 mM Na_3_VO_4_, 1 mM phenylmethylsulfonyl fluoride and 10 µg/ml each of aprotinin, pepstatin and leupeptin). Dishes were shaken for 30 min on ice. Lysates were vortexed, sonicated and cleared by centrifugation (5 min, 16,000 ***g***, 4°C). Supernatants were incubated for 1 h at 4°C with 10 µl GFP Trap_M, which contains a recombinant fragment of alpaca anti-GFP-antibody covalently coupled to the surface of paramagnetic beads (ChromoTek, Martinsried, Germany). The beads were washed three times with washing buffer (50 mM Tris-Cl pH 7.5, 150 mM NaCl, 2 mM EDTA, 0.1% Igepal CA-630) and split in two aliquots. One aliquot was used for radiometric assay to determine kinases activities with the peptide substrate DYRKtide as described (Abu Jhaisha et al., 2017). The other aliquot was eluted using Laemmli sample buffer at 95°C for 5 min for immunoblot analysis to assess the relative tyrosine phosphorylation of the DYRK1A constructs. Samples were excluded from analysis if the expression of the variant construct differed from wild type by more than a factor of 10. This criterion was established during data analysis in order to manage unexpected failure of expression in individual samples.

### Western blotting

Proteins were separated by SDS-PAGE and blotted onto nitrocellulose membranes (Amersham Protran 0.45 μm NC, GE Healthcare, Pittsburgh, PA, USA). Membranes were blocked using 3% bovine albumin in TBST (10 mM Tris-Cl pH 7.5, 100 mM NaCl plus 0.1% Tween-20) and incubated with primary antibodies overnight at 4°C. Incubation with horseradish peroxidase (HRP)-conjugated secondary antibodies was done at room temperature for 1 h, followed by washing with TBST. Chemiluminescence signals were detected using LAS-300 Imaging system (FujiFilm, Düsseldorf, Germany) and densitometrically evaluated with the help of Aida Image Analyzer 3.52 software (Raytest, Straubenhardt, Germany).

### *In vitro* translation

A reconstituted *E. coli*-based *in vitro* transcription-translation system (PURExpress, New England Biolabs, Beverley, MA, USA) was used to express a DYRK1A(28-499) construct fused to an N-terminal Strep-tag 2 sequence. Reactions were incubated in a volume of 10 μl with 10 ng/μl plasmid DNA at 37°C for 90 min. Autophosphorylation of Y321 in DYRK1A was assessed by immunoblot blot with rabbit anti phospho-HIPK2 (Tyr361) antibody. Band intensities were normalized to total protein levels as detected by Strep-Tactin conjugated to HRP (IBA Life Sciences, Göttingen, Germany).

### Thermal stability assay

The assay was developed based on the protocol of cellular thermal shift assay (CETSA, Jafari et al., 2014) with modifications. Stable HEK293 cells expressing wild type GFP-DYRK1A or the L295F mutant were harvested by scraping and washed with PBS at room temperature. Cells from one 10-cm dish were resuspended in 1 ml PBS supplemented with protease inhibitors. Cell suspensions were divided into 100 µl aliquots in thin-walled micro test tubes. Samples were incubated for 3 min in a thermocycler at varying temperatures as indicated and subsequently kept at 25°C for a further 3 min before snap freezing in liquid nitrogen. Different from the original method, cells were lysed with the help of a non-ionic detergent, because a significant portion of cellular DYRK1A molecules is thought to be associated with the cytoskeleton or chromatin fraction. After adding Triton X-100 to a final concentration of 0.1%, cells were incubated on ice for 15 min before soluble fractions were separated from precipitated proteins by centrifugation (20 min, 20,000 ***g*** at 4°C). The abundance of DYRK1A in the soluble fraction was quantified by western blotting.

### Fluorescence microscopy

COS7 cells cultured on coverslips in 12-well plates were transiently transfected with different expression constructs and incubated for 48 h. The cells were washed with PBS, fixed in 4% paraformaldehyde for 20 min and quenched with 50 mM NH_4_Cl in PBS for 5 min. All incubations were done at room temperature. After washing with PBS, the coverslips were mounted onto slides using Vectashields Mounting Medium (Vector Laboratories, Burlingame, CA, USA) containing 1.5 µg/ml 4′,6-diamidino-2-phenylindole (DAPI) for nuclear staining. Images were obtained with a Zeiss LSM 710 confocal microscope (Zeiss, Jena, Germany) using the C-apochromat 63×/1.20 water objective of Zeiss. GFP was excited with the 488 nm line of an argon laser and DAPI was excited with 405 nm laser diode. Images were analyzed with the Zen 2012 software (Zeiss, Jena, Germany).

### Visualization of DYRK1A structure

Molecular visualization and analysis of the kinase domain structure of DYRK1A (PDB entry 2VX3; Soundararajan et al., 2013) and the illustration of the positions of the missense mutations and the pY321 residue was performed by BallView 1.4 Software (Moll et al., 2006).

### Statistics

The one-sample *t*-test (two-tailed) was applied to test the hypothesis that readout parameters were different between wild-type DYRK1A and a particular missense variant (GraphPad PRISM, Graphpad Software Inc.). Samples sizes (*n*) reflect independent experiments.

## Supplementary Material

First Person interview
