# Supplementary material for: Functional characterization of DYRK1A missense variants associated with a syndromic form of intellectual deficiency and autism
Source: Biol Open. 2018 Apr 15;7(4):bio032862. doi: 10.1242/bio.032862 (PMC5936063; doi:10.1242/bio.032862)
Supplement: First Person interview [file biolopen-7-032862-s1.pdf]

© 2018. Published by The Company of Biologists Ltd | Biology Open (2018): doi:10.1242/bio.032862: Supplementary information

FIRST PERSON

# First person – Esti Wahyu Widowati

First Person is a series of interviews with the first authors of a selection of papers published in Biology Open, helping early-career researchers promote themselves alongside their papers. Esti Wahyu Widowati is first author on 'Functional characterization of DYRK1A missense variants associated with a syndromic form of intellectual deficiency and autism', published in BiO. Esti is a PhD student in the lab of Walter Becker at RWTH Aachen University, Aachen, Germany, investigating molecular characterization of the DYRK family of protein kinases.

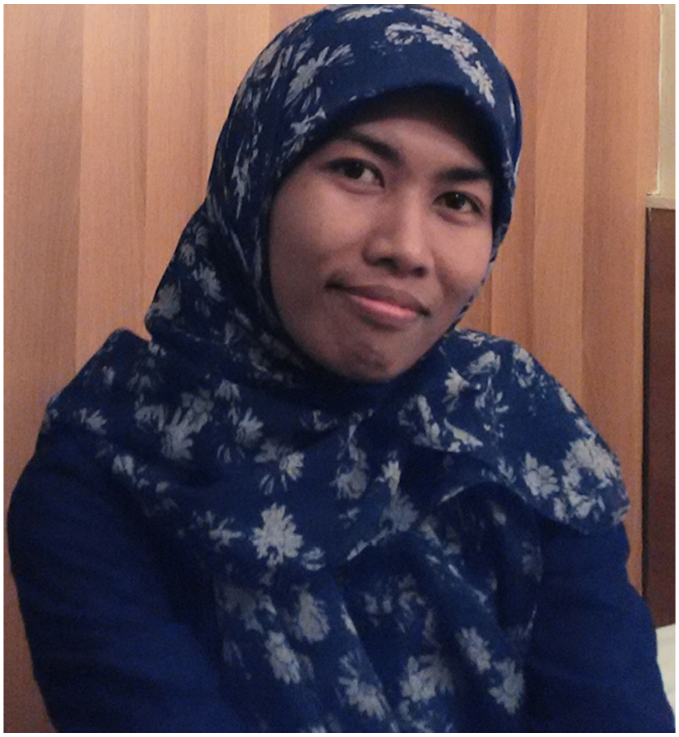

Esti Wahyu Widowati

**What is your scientific background and the general focus of your lab?**

I studied organic chemistry during my undergraduate and graduate studies. Then my scientific career led me to study biotechnology, which gave me insight into some basic concepts in molecular biology. I started my PhD at the Institute for Pharmacology and Toxicology, RWTH Aachen University, under the guidance of Prof. Walter Becker, where I learned about the DYRK family of mammalian protein kinases. I am studying DYRK1A, focusing on its biochemical and functional characterization.

**How would you explain the main findings of your paper to non-scientific family and friends?**

The function of DYRK1A is highly dependent on its gene dosage. Decreased levels of DYRK1A resulting from a mutation in one of the two copies of the gene is a cause of mental retardation autosomal dominant 7 (MRD7), a disease characterized by microcephaly, intellectual disability, epileptic seizures, autism spectrum disorder and language delay. Patients with MRD7 have been identified to have a variety of genetic mutations, including missense variants of DYRK1A. In a missense mutation, there is a change in one DNA base pair, resulting in the replacement of one amino acid. What is interesting about these mutations is that the amino acid substituted during this mutation might be important for the kinase activity of the protein. In our cell culture experiments, we generated DYRK1A mutants and compared their activity and biochemical properties with the non-mutated form of DYRK1A.

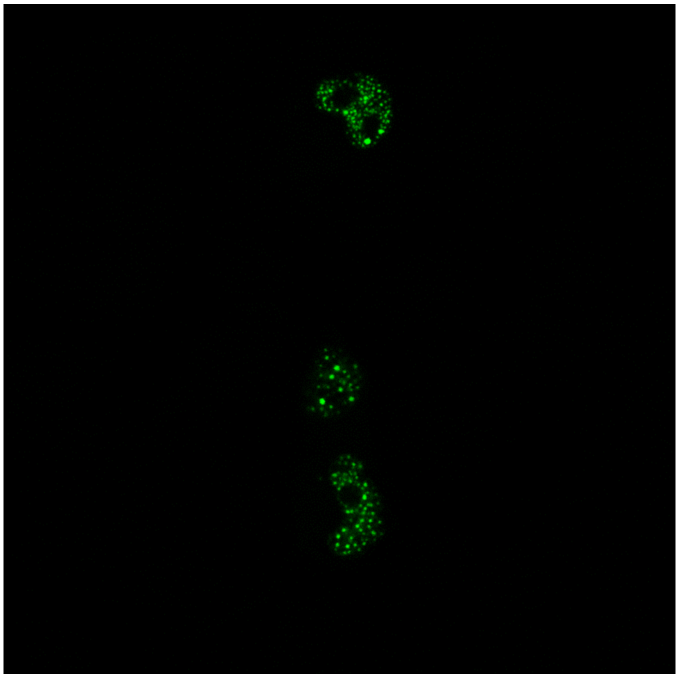

Subcellular localization of wild-type DYRK1A expressed in COS7 cells

Esti Wahyu Widowati's contact details: Institute for Pharmacology and Toxicology, RWTH Aachen University, Aachen 52074, Germany.
E-mail: ewidowati@ukaachen.de

**"The results of this study contribute to the understanding of the structure–function relationship of protein kinases, mainly the DYRK1A sub-family."**

**What are the potential implications of these results for your field of research?**

Previous research has shown that mutations in the catalytic domain of DYRK1A (K188R and D287N) result in catalytically inactive DYRK1A. We found that five other missense mutations

(L245R, F308V, S311F, S346P and R467Q) also eliminate tyrosine phosphorylation and another variant (L295F) had a more subtle effect on protein function. The results of this study contribute to the understanding of the structure–function relationship of protein kinases, mainly the DYRK1A sub-family.

### What has surprised you the most while conducting your research?

Amino acid substitution in the catalytic domain of DYRK1A protein kinase was expected to eliminate tyrosine phosphorylation. However, one mutant located in catalytic domain (L295F) shows a comparable tyrosine phosphorylation to the wild type.

### What changes do you think could improve the professional lives of early-career scientists?

The huge competition to get a research grant is the main problem for early-career scientists. To be more focused on developing their research and getting funding, early-career scientists should be given more time in the lab rather than teaching classes and doing so much paperwork.

### What's next for you?

Completing my PhD and looking for any possibility for doing postdoctoral research.

#### Reference

**Widowati, E. W., Ernst, S., Hausmann, R., Müller-Newen, G. and Becker, W.** (2018). Functional characterization of DYRK1A missense variants associated with a syndromic form of intellectual deficiency and autism. *Biol. Open* **7**: bio032862, doi:10.1242/bio.032862.
